# Precursors to Natural Grammar Learning: Preliminary Evidence from 4-Month-Old Infants

**DOI:** 10.1371/journal.pone.0017920

**Published:** 2011-03-22

**Authors:** Angela D. Friederici, Jutta L. Mueller, Regine Oberecker

**Affiliations:** Max Planck Institute for Human Cognitive and Brain Sciences, Leipzig, Germany; University of Oxford, United Kingdom

## Abstract

When learning a new language, grammar—although difficult—is very important, as grammatical rules determine the relations between the words in a sentence. There is evidence that very young infants can detect rules determining the relation between neighbouring syllables in short syllable sequences. A critical feature of all natural languages, however, is that many grammatical rules concern the dependency relation between non-neighbouring words or elements in a sentence i.e. between an auxiliary and verb inflection as in *is* sing*ing*. Thus, the issue of when and how children begin to recognize such non-adjacent dependencies is fundamental to our understanding of language acquisition. Here, we use brain potential measures to demonstrate that the ability to recognize dependencies between non-adjacent elements in a novel natural language is observable by the age of 4 months. Brain responses indicate that 4-month-old German infants discriminate between grammatical and ungrammatical dependencies in auditorily presented Italian sentences after only brief exposure to correct sentences of the same type. As the grammatical dependencies are realized by phonologically distinct syllables the present data most likely reflect phonologically based implicit learning mechanisms which can serve as a precursor to later grammar learning.

## Introduction

Children are able to learn languages spontaneously within just a few years. To do so, infants must be equipped with remarkable language learning abilities. The ability to extract relations between adjacent syllables (AB) from auditory input on the basis of the statistical computation of transitional probabilities between the elements A and B may be present from birth [1], and has clearly been evidenced at 7 to 8 months of age [2]–[6]. Data from a behavioural study suggest that at this age, infants' learning might even go beyond a calculation of transitional probability, and possibly also involving the extraction of abstract rules from three-syllable sequences [3]. Thus, the ability to extract and generalize abstract rules between adjacent elements in highly predictive sequences [1]–[6] is present very early in life. This early ability may be based on young infants' sensitivity to acoustic-phonological regularities in the auditory input. Event-related brain potential (ERP) studies provide evidence of phonological sensitivities at a very early age. The ability to discriminate between different phonemes embedded in syllables and statistical relations between syllables [2], [7]–[9] can be observed in newborns. Effects of language-specific ordering of stressed and unstressed syllables in 2-syllable words following input regularities were reported for 4-month-old infants exposed to French and German, respectively [10]. This latter study indicates that the dependency between adjacent elements and their regularity is detected as a result of natural language input at the age of 4 months. The grammar of every natural language, however, does not only require the recognition of dependencies between adjacent elements, but moreover between non-adjacent elements in a sentence.

The learning of these non-adjacent dependencies is much more difficult than learning adjacent dependencies. The relation between the elements A and B with an intervening variable X element (as in *is* sing*ing* ) can only be recognized when abstracting over X (i.e. the verb), thereby disregarding local transitional probabilities for the sake of distant relations. There are a number of behavioural studies which investigated learning of non-adjacent dependencies, both in artificial learning experiments and in natural language acquisition. These studies used different paradigms, while artificial grammar learning involved testing after very brief familiarization periods [4], [6], [11] studies on natural grammar acquisition tested non-adjacent dependencies in the infants' target language [12], [13]. Both types of studies suggest that the learning of non-adjacent dependencies occurs around the age of 17-to-18 months [4], [11], [12], [13]. In artificial grammar learning studies infants at the age of 17 months were shown to be able to extract the relation between A and B in an AXB structure from 3-syllable strings when the variability of the X-element is high [4]. This learning effect is present in 17-month-olds, but not in 12- or 15-month-olds [11]. In these learning experiments the training lasted approximately 3 minutes, before testing took place. In studies investigating natural language acquisition, it was shown that 18-month-old infants learning English as their native language were able to track the relationship between *is* and *ing* in phrases such as *is* digg*ing* (versus *can* digg*ing*), but that 15-month-olds could not [13]. Also, it was reported that German-learning 19-month-olds were able to recognize non-adjacent dependencies but only under the condition that the intervening word was clearly marked by a fixed morphosyntactic element like, for example, the suffix –*ly* marking adverbs in English [12]. Moreover, it has been shown that the ability to learn non-adjacent dependencies at this age is modulated by the distributional probabilities in the ambient natural language input [14]. Thus, these studies indicate that the ability to learn non-adjacent dependencies from input in a given language appears to be present relatively late during development.

The goal of the present study was to investigate whether infants at a younger age can track non-adjacent dependencies (i.e. the relation between A and B in AXB structures) in a novel natural language in a learning experiment with controlled language input. Here we used ERP as the dependent variable, as it allowed us to test for learning effects independent of the infant's behaviour. We decided to test 4-month-old infants since prior research suggested early verbal memory and phonological discrimination capacities to be present at this age. First, in behavioural studies it was shown that 2-month-olds are able to detect word order changes between two sentences [15]. Second, brain imaging work demonstrated that 3-month-olds are sensitive to sentence repetition with a delay of 14 seconds suggesting early verbal memory capacities [16]. Third, prior ERP research had shown that infants at the age of 4 months are sensitive to phonological regularities of adjacent syllables as results of their natural language input [10]. Based on these data we hypothesised that 4-month-old German infants might be able to learn grammatical regularities of non-adjacent elements possibly on the basis of their inherent phonological properties.

**Figure 1:**
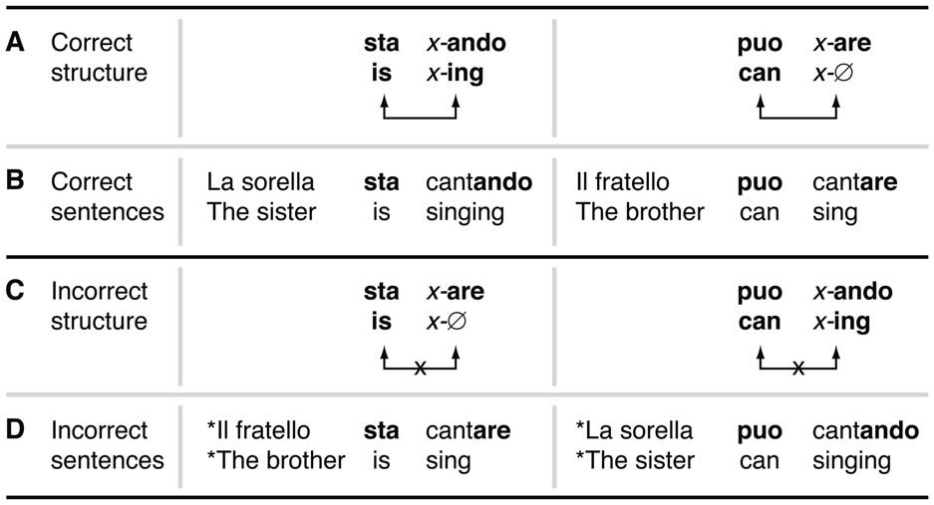
Structure and examples of Italian stimulus sentences. The figure displays the grammatical dependency between the auxiliaries (*sta*/*is* and *puo*/*can*) and the respective Italian verb inflections (-*ando* and -*are*). (A) Correct grammatical relation between *sta* and -*ando* as well as *puo* and -*are* with *x* as a place holder for the verb stem. (B) Correct example sentences for the structure represented in (A). (C) Incorrect grammatical relation between *sta* and -*are* as well as *puo* and -*ando* with *x* as a place holder for the verb stem. (D) Incorrect example sentences for the structure represented in (C). Relation between crucial non-adjacent elements is indicated by arrows. An asterisk indicates an incorrect sentence.

Here German native infants were exposed to stimulus material consisting of four-word correct Italian sentences containing systematic, rule-based non-adjacent dependencies between two elements i.e. between an auxiliary and a verb suffix, similar to the auxiliary *is* and the suffix –*ing* in *is X-ing* (see Figure 1). As all infants grew up in monolingual German-speaking environment, therefore, the Italian sentences were completely novel to them.

**Figure 2:**
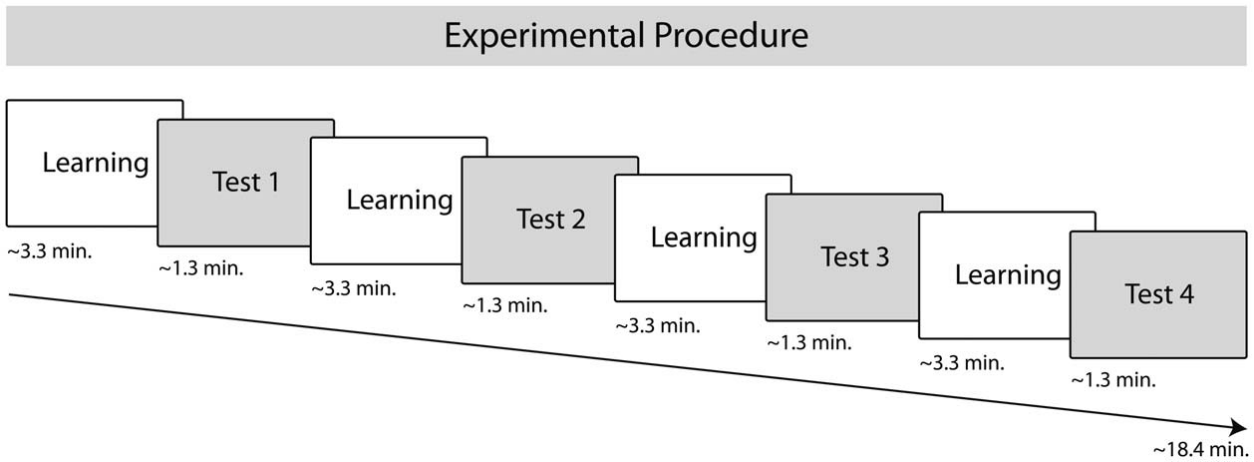
Experimental procedure. The experimental procedure consisted of short learning and test phases: Learning phase approx. 3.3 minutes (containing 64 correct sentences), Test phase approx. 1.3 minutes (containing 8 correct and 8 incorrect sentences). The experiment consisted of 4 learning and 4 test phases.

The correct Italian sentences with which the German infants were familiarized contained two different non-adjacent dependency types between the auxiliary and the verb suffix (in italics): namely *sta X-ando* (*is X-ing*) as in *sta* cant*ando/is* sing*ing* and *può X-are* (*can X*-**Ø**) as in *può cantare/can* sing. The variable element X was realized in the experiment by inserting 32 different verb stems. All correct sentences contained either the *sta X-ando/is X-ing* or the *può X-are/can X-* **Ø** structure. Each of these structures were either preceded by a masculine (*il fratello/the brother*) or a feminine (*la sorella/the sister*) noun phrase (for examples see Figure 1. During four learning phases, each with 64 sentences, lasting approximately 3.3 minutes, infants were familiarized with a total of 256 correct sentences (4×64 correct sentences) resulting in an overall familiarization time of 13.2 minutes (for details see Materials and Methods). Each learning phase was immediately followed by a test phase (see Figure 2).

During the test phases, incorrect sentences were presented together with correct sentences. A total of 128 incorrect sentences were constructed by interchanging the auxiliary and the respective corresponding suffix. Both correct and incorrect sentences were created using a cross-splicing procedure. Across all 4 test phases, 32 novel correct sentences (16 *sta X-ando* and 16 *può X-are*) and 32 incorrect sentences (16 *sta X-are* and 16 *può X-ando*) were played to the infants (for details see Materials and Methods).

Our stimulus material was specifically designed to avoid phonological differences between verbs across the correct and incorrect non-adjacent dependency conditions. We had used two A..B frames, *sta…ando* and *puo…are*, for the correct condition and two frames, *puo…ando* and *sta…are*, for the incorrect condition. The verbs (both stems and suffixes) were identical across correct and incorrect conditions which was ensured by the cross-splicing technique that we used. This procedure ensured that acoustically identical material was tested in the correct and in the incorrect condition and that any difference observed between the conditions must attributed to the learning of the relationship between auxiliary and verb suffix.

Thus, the present stimulus material with fixed syntactic frames containing 2 dependencies with 32 intervening X-elements, provides an interesting comparison to that used in the study by Gomez and colleagues [4], [11] which contained 3 dependencies with 24 intervening X-elements. A clear difference between these earlier behavioural studies and the present ERP study is the familiarization or learning time. While the familiarization phase lasted approximately 3 minutes in the earlier behavioural studies, we used a paradigm with four learning phases leading to an overall learning time of 13.2 minutes, which was separated by four test phases in which correct and incorrect items were presented.

The present study set out to test whether 4-month-old infants could learn non-adjacent dependencies in a novel language in the above described repetitive learning paradigm. Tests of learning success were applied after each of the four learning phases. During the test phases we recorded event-related brain potentials (ERPs) while the German infants were listening to correct and incorrect Italian sentences after exposure to correct sentences during the learning phases. Crucial for our hypothesis was to observe a general effect of grammatical learning (correct vs. incorrect) across all test phases as such an effect would provide evidence for successful learning of non-adjacent dependencies in a novel language (German infants learning Italian). Of secondary interest was to see whether successful learning would occur already after first learning phase of 3.3 minutes that is in the first test phase or only later during the experiment that is after an overall learning time of 13.2 minutes evidenced in the last test phase. Based on the prior behavioural artificial grammar experiments [11] we expect to see grammatical learning in the last test phase, rather than in the first test phase.

## Results

**Figure 3:**
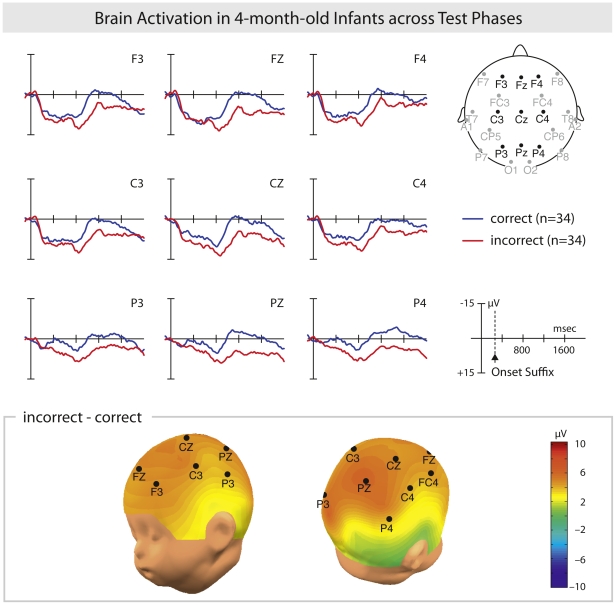
The grammaticality effect. Top: Grand average event-related potentials of 4-month-old infants (n = 34) for the processing of the verb averaged across the four test phases. The processing of the incorrect condition (red line) is plotted against the processing of the correct condition (blue line). The solid vertical line indicates the onset of the verb, the broken vertical line at the scale plot indicates the onset of the suffix. Negative is plotted upwards. Bottom: Isovoltage map showing the scalp distribution of the effect. Positive difference is colour-coded in red.

First, in order to test for a general grammatical learning effect the ERPs in response to the verb and its suffix were averaged across the four test phases separately for correct and incorrect sequences. Figure 3 displays the grand averages across all test phases of 34 infants' brain responses and their scalp distribution. In response to grammatically incorrect compared to correct sentences, the ERPs indicated a clear grammatical learning effect with a more positive-going wave with a centro-parietal distribution (see Figure 3). The statistical analyses were computed for different time windows (TW) (see Material and Methods). The positivity was significant between 900–1300 ms after verb onset, that is, between 640 and 1040 ms after the onset of the verb's suffix as indicated by a significant Condition effect (correct/incorrect) for TW5 (F(1,33) = 8.31, p<.01, ω^2^ = 0.1075) and TW6 (F(1,33) = 4.68, p<.05, ω^2^ = 0.0541). The present data give a clear neurophysiological indication of discrimination between correct and incorrect grammatical sentences, and thereby provide evidence of learning of the non-adjacent dependency relation in a language unknown to the infants prior to the experiment.

Second, in order to test whether learning had occurred between the first and the last testing phase an ANOVA including the first Test Phase and the last Test Phase with the factors Condition (correct/incorrect) and Test Phase (TP1/TP4) was conducted. The analysis revealed a trend for a Test Phase×Condition interaction between 1100–1300 ms (TW6) post verb onset (F(1, 33) = 3.69, p = .06, ω^2^ = 0.0197). No other effect was found. Subsequent separate analyses for TP1 and TP4 in this time window revealed a significant effect for the last test phase (TP4: F(1,33) = 5.17; p<.03; ω^2^ = 0.0613), but not for the first test phase (TP1: F(1,33) = 0.00; p = .99).

**Figure 4:**
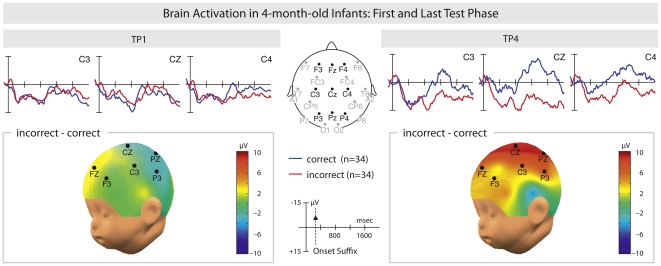
The learning effect. Grand averages of event-related potentials of 4-month-old infants (n = 34) for the processing of the verb left: grand averages for first test phase (TP1), right: grand averages for last test phase (TP4). The processing of the incorrect condition (red line) is plotted against the processing of the correct condition (blue line). The solid vertical line indicates the onset of the verb, the broken line at the scale plot indicates the onset of the suffix. Negative is plotted upwards.

This latter analysis indicates that the infants in Test Phase 1 after the first training phase did not yet differentiate between correct and incorrect sentences at the beginning of the experiment, but that they did in Test Phase 4. This difference between Test Phase 1 and 4 clearly shows that infants improved learning the non-adjacent dependencies during the experiment (see Figure 4).

## Discussion

The present data demonstrate that 4-month-old infants can extract dependencies between non-adjacent elements in sentences from brief exposure to a natural, non-native language. This ability is reflected in a grammatical learning effect in the form of a more positive going wave for grammatically incorrect compared to grammatically correct dependency relations. The emergence of the sensitivity to the grammatical regularities indicates that infants extracted the dependencies within the two pairs of non-adjacent elements (i.e. the auxiliaries and the respective verb suffixes) from correct sentences they had heard during the training phases. Learning of these dependencies could be based on different types of phonological cues marking the crucial elements in Italian. Infants may have both extracted and stored the full phonological forms of the related auxiliary and suffix (*sta* X-*ando*, *può* X-*are*) in their memory or they may have extracted partial phonological information contained in the crucial elements, such as vowel quality in the different elements (-*a* X-a–o, -*ò* X-a–e). As an additional possibility it has to be considered whether the observed positivity to incorrect sentences could be based on the phonological repetition of same or similar vowels in the auxiliary and the suffix in the test items alone (i.e. -*a* X *a* in *sta* X *are*, and –*uò* X *o* in *può* X *ando*). Although repetition is likely to be processed by infants from early on [7]–[10] it is not informative in our study as a single cue as repetition of vowels occurs both in correct, as well as in incorrect sentences (correct: -a X –a in sta X-ando, incorrect: -*ò* X –o in pu*ò* X-ando). Further, in the infant ERP literature, increased brain responses have been observed to phonological deviances in phoneme and syllable sequences rather than to repetition [7]–[10]. These studies have used so-called standard oddball paradigms in which a particular phoneme or syllable is repeated several times (frequent stimulus) before a deviant (infrequent) stimulus is presented. Increased brain responses have been observed to the deviant stimulus either in form of a positivity between 200 and 400 ms [8]–[10] or a small negativity peaking around 50 ms followed by a positivity [7]. Repetition of the same stimulus, in contrast, was found to lead to a decrease of the ERP amplitude [8]. Since repetition leads to an amplitude decrease, the present positivity cannot be explained as a simple stimulus repetition effect. The only alternative interpretation would be to view the positivity as a memory-based deviance effect including the possibility that specific repetition or change patterns among vowels were memorized. During the training phases only correct auxiliary-suffix combinations were heard making the correct combinations overall more frequent than the incorrect combinations which served as the basis for memory formation. Such a memory-based deviance effect has been reported earlier as a result of language specific input frequencies of adjacent syllables in 4-month-old-infants [10]. If the memory-based interpretation for the present data is valid, this would mean that infants did learn phonological aspects of non-adjacent elements of frequent versus less frequent combinations across the experiment. The plain assumption that infants initially somehow preferred either the correct or the incorrect auxiliary-suffix combination over the respective other is unlikely, since no significant difference between the two conditions was found in Test Phase 1. Thus the present data suggest that infants must have recognized and memorized the systematic relation between the two respective non-adjacent elements independent of the intervening verb (X). It is conceivable in the present stimulus material that the positional salience of the suffix in sentence final position may have eased the detection of one of the crucial elements of the dependency relation [17], as this cue has been proposed to facilitate the computation of syntactic structures in older children [18], [19].

Interestingly, the grammaticality effect observed for 4-month-olds in the present study is neurophysiologically established as a positivity. In adults and older children, late positivities at the sentence level usually mark syntactic processes [20]–[22]. Italian adults who were tested with the stimulus material used in the present study demonstrated a similar centro-parietal positivity which followed a widely distributed negativity (N400) [23]. The N400 has been related to processes concerning word form and meaning [23], [24]. The positivity in Italian native speakers was seen as an instance of a P600, reflecting syntactic processes [23]. This stands in clear contrast to German adult second language learners tested with the same material, who showed only the lexical N400 effect and a very different frontally distributed short-lasting positive component that was classified as a P3a component, reflecting general attention-based cognitive processes when being confronted with task-relevant novel stimuli. Thus, adult learners were only able to learn the non-adjacent dependencies from the Italian sentence input when their attention was explicitly directed to the rule extraction.

The similarity of the ERP effect in 4-month-old German infants to adult native speakers and its dissimilarity to German adult second language learners tentatively suggests that native learning may be restricted to a sensitive time window during development. It is conceivable that this ability is initially based on implicit associative learning mechanisms which are most successful during early development when the prefrontal cortex does not yet exhibit cognitive control [25], [26].

The current finding that very young infants are able to extract non-adjacent grammatical dependencies from the auditory input is of great interest, as this ability is a fundamental prerequisite to the acquisition of complex syntax in every human language [27], [28]. The grammaticality effect observed indicates that the ability to extract rule-based dependencies between non-adjacent elements in sentences of a novel language can already be observed in 4-month-old infants, thereby providing a precursor for later grammar learning.

## Material and Methods

### Participants

Seventy-four 4-month-old (+/−7 days; 39 male) monolingual infants growing up in German-speaking families participated in the present study. Ethical approval was obtained from the ethics committee of the Charité-Universitätsmedizin Berlin, and parents gave written informed consent for their children's participation in the study. From the 74 infants six had to be excluded due to technical problems and 34 as they did not reach the required criteria, meaning data from a total of 34 infants were included in the final analysis. To be included children had to have 8 accepted trials in the correct condition and 8 in the incorrect condition within the four test phases and had to pass Test Phase 1 and Test Phase 4. Furthermore, mean number of trials Test Phase 1 and Test Phase 4 was about 5 trials (TP1: correct = 5.36, incorrect = 5.38; TP4: correct = 4.71, incorrect 4.71).

### Stimuli

The Italian sentences used in the present study contained either the masculine definite determiner *il* or the feminine definite determiner *la*, 2 animate nouns, namely *fratello* (brother) or *sorella* (sister), 2 different auxiliaries (*può*, to be able to, 1^st^ person singular or *sta*, to be, 1^st^ person singular), and 32 verbs occurring either in the infinitive (*e.g. cantare*/to sing) or in the progressive form (*e.g. cantando*/singing). Mean length of the verb stems was 260 ms, verb stem plus *–are* 452 ms and verb stem plus –*ando* 530 ms. Additional acoustic analyses of the stimulus material were conducted for the verb stem and the suffix to make sure that the verb stem was not unstressed and thereby less salient. These analyses were conducted for acoustic intensity and pitch. The statistics for intensity revealed that the average maximum of the verb stem was higher that that of the verb suffix (78,08 vs. 76,95 dB). This difference was statistically significant (t(1,126) = 2,602, p = .01). Maximal pitch values between verb stem and verb suffix did not differ significantly (330,41 vs 336,22 Hz (t(1,126) = −,845, p = .4). These measures show that the verb stems are acoustically more salient than their suffixes.

Within the sentences, a non-adjacent dependency existed between the auxiliary and the suffix of the following verb: the auxiliary *può* required the infinitive verb form (i.e. *X-are*) whereas the progressive form (i.e. *X-ando*) was needed after the occurrence of the auxiliary *sta* (e.g. *la sorella può cantare*, *la sorella sta cantando*). In total, 128 different correct sentences were created (2 noun phrases×2 auxiliary-verb inflection combinations×32 verbs). In contrast to correct sentences, incorrect sentences included a wrong combination between the auxiliary and the following verb form (i.e. *può X-ando*, *sta X-are*).

Both the correct and incorrect sentences were generated in the same manner using a cross-splicing procedure, exchanging the verb with the verb from a different sentence. Cross-splicing was used in both conditions to avoid any possible acoustic difference between correct and incorrect sentences. This is because natural production of incorrect sentences could have led to syllable lengthening and thus to additional prosodic cues in the incorrect condition.

All sentences were spoken with a sentence intonation by a female native speaker of Italian and digitally recorded. A total of 96 correct sentences were created. For each subject, 64 of these 96 correct sentences were chosen for the learning phases and 32 for the test phases. During each learning phase, all 64 correct sentences were presented (256 (4×64) correct sentences in all 4 learning phases) in pseudo-randomized order. The remaining 32 of the 96 correct sentences and 32 corresponding grammatically incorrect sentences occurred during the test phases. Each of the 4 test phases consisted of 8 correct and 8 incorrect sentences.

### Procedure

Babies and caregivers were seated in a soundproof booth. Infants were either placed on the caregiver's lap or laid in a safety seat. In order to minimize eye movements, a silent video was presented while the sentences were presented via loudspeakers. The experiment consisted of 4 alternate learning and test phases, starting with a learning phase and ending with a test phase. Each learning phase lasted approximately 3.3 minutes. No pauses were inserted between the different phases. In order to minimize the duration of the entire experiment, we used different inter-stimulus-intervals (ISIs) from sentence onset to the onset of the following sentence in the learning phases and in the test phases. In the learning phases, the ISI was 3000 ms, while it was 5000 ms in test phases.

### Data recording and analysis

The EEG was continuously recorded from Ag/AgCl electrodes at sites F7, F3, FZ, F4, F8, FC3, FC4, T7, C3, CZ, C4, P7, CP5, CP6, T8, P3, PZ, P4, P8, O1, O2, A1 and A2 (according to the 10–20 International System of Electrode Placement). The electrodes were secured in an elastic electrode cap (Easy Cap, Falk Minow) and the ERP electrodes were referenced to CZ during recording. Electrooculograms (EOG) were recorded bipolar supraorbital and infraorbital to the right eye (V−, V+) as well as from electrodes located lateral to the left and to the right eye (H−, H+). The electrode impedances were mostly kept below 10 kΩ, and always below a maximum of 15 kΩ. The electrical signals were digitized with a sampling rate of 500 Hz. The EEG was algebraically re-referenced to the average of both mastoids (A1, A2). A zero-phase digital band-pass filter ranging from 0.3–20 Hz (−3 dB cut-off frequencies of 0.38 and 19.91 Hz) was used to remove drifts and muscle artefacts from the EEG while still preserving most of the original signal. In the following step, trials exceeding a standard deviation of 80 µV within a sliding window of 500 ms were rejected automatically. In the present analyses, we included only children who met the criteria of 8 trials per condition and had at least one trial within the first and the last test phase. All other infants were excluded from further analyses. Event-related brain potentials were evaluated for each participant during the test phase in both conditions for 2000 ms time-locked to the onset of the critical verb with a 100 ms pre-stimulus baseline.

In order to investigate the grammaticality effect for all test phases, an ANOVA with the factors Condition (correct/incorrect) and Electrode Site (F3, FZ, F4, C3, CZ, C4, P3, PZ, P4) was conducted. To establish the learning effect during the experiment, an ANOVA with the factors Condition, Electrode Site and Test Phase (TP1/TP4) was conducted. The following time windows (TW), time-locked to the verb onset, were analyzed: 100 to 300 ms (TW1), 300 to 500 ms (TW2), 500 to 700 ms (TW3), 700 to 900 ms (TW4), 900 to 1100 ms (TW5), 1100 to 1300 ms (TW6), 1300 to 1500 ms (TW7), 1500 to 1700 ms (TW8) and 1700 to 1900 ms (TW9). Data in Figures 3 and 4 are displayed with a timescale indicating the verb onset (i.e. the point at which the sentence material was spliced). For all ANOVAs, the Greenhouse-Geisser correction was applied whenever there was more than one degree of freedom. Effect size was calculated according to Cohen [29].
